# Complement C4 Is Reduced in iPSC-Derived Astrocytes of Autism Spectrum Disorder Subjects

**DOI:** 10.3390/ijms22147579

**Published:** 2021-07-15

**Authors:** Fernanda Mansur, André Luiz Teles e Silva, Ana Karolyne Santos Gomes, Juliana Magdalon, Janaina Sena de Souza, Karina Griesi-Oliveira, Maria Rita Passos-Bueno, Andréa Laurato Sertié

**Affiliations:** 1 Centro de Pesquisa Experimental, Hospital Israelita Albert Einstein, 05652-900 São Paulo, Brazil; fernanda_mansur@yahoo.com.br (F.M.); andre.teles@einstein.br (A.L.T.e.S.); ana.ksgomes@einstein.br (A.K.S.G.); juliana.magdalon@einstein.br (J.M.); karina.griesi@einstein.br (K.G.-O.); 2 Faculdade Israelita de Ciências da Saúde Albert Einstein, 05521-200 São Paulo, Brazil; 3 Laboratório de Endocrinologia e Medicina Translacional, Departamento de Medicina, Universidade Federal de São Paulo, 04021-001 São Paulo, Brazil; ninasenasouza@gmail.com; 4 Departamento de Genética e Biologia Evolutiva, Instituto de Biociências, Universidade de São Paulo, 05508-090 São Paulo, Brazil; passos@ib.usp.br

**Keywords:** complement system, autism spectrum disorder, iPSC-derived astrocytes, complement C4, synaptic pruning

## Abstract

In recent years, accumulating evidence has shown that the innate immune complement system is involved in several aspects of normal brain development and in neurodevelopmental disorders, including autism spectrum disorder (ASD). Although abnormal expression of complement components was observed in post-mortem brain samples from individuals with ASD, little is known about the expression patterns of complement molecules in distinct cell types in the developing autistic brain. In the present study, we characterized the mRNA and protein expression profiles of a wide range of complement system components, receptors and regulators in induced pluripotent stem cell (iPSC)-derived neural progenitor cells, neurons and astrocytes of individuals with ASD and neurotypical controls, which constitute in vitro cellular models that recapitulate certain features of both human brain development and ASD pathophysiology. We observed that all the analyzed cell lines constitutively express several key complement molecules. Interestingly, using different quantification strategies, we found that complement C4 mRNA and protein are expressed in significantly lower levels by astrocytes derived from ASD individuals compared to control astrocytes. As astrocytes participate in synapse elimination, and diminished C4 levels have been linked to defective synaptic pruning, our findings may contribute to an increased understanding of the atypically enhanced brain connectivity in ASD.

## 1. Introduction

The complement system, which plays crucial roles in the innate defense against pathogens and damaged cells [1], has been increasingly implicated in brain development and plasticity [2,3]. All major cell types in the brain have been shown to constitutively express at least some components, receptors and regulators of the three pathways of complement activation (classical, lectin and alternative), which contribute to some key cellular processes in the developing brain, including neurogenesis [4], neuronal migration [5] and synapse pruning and remodeling [6,7,8,9]. Consequently, inappropriate expression or activation of the complement system might alter the establishment of precise neural networks in the brain during development, resulting in cognitive dysfunction. Indeed, a growing number of studies has associated dysregulation of specific components of the complement system with neurodevelopmental disorders [10], such as schizophrenia [8] and autism spectrum disorder (ASD) [11,12].

ASD is a heterogeneous neurodevelopmental condition, characterized by deficits in social interaction and the presence of repetitive, restricted behaviors [13]. The etiology of ASD is very complex and may involve genetic, epigenetic, environmental and immunological risk factors [14,15]. Previous studies have found a potential association between abnormal expression of complement system elements and ASD risk. Genetic variants in the complement *C4B* gene resulting in non-expression of the gene (*C4B* null alleles) were associated with increased risk for this disorder [16,17,18]. Altered levels of complement proteins, such as C4, C1q, C3, C5 and CFI, were observed in the blood of individuals with ASD [19,20,21,22]. Finally, the expression patterns of several complement genes, such as *C1q*, *C2*, *C3*, *C4*, *MASP1* and *CR3*, were found to differ in post-mortem brain tissue samples from ASD subjects compared to control subjects [11,12].

However, the expression profile of complement genes and proteins during the initial stages of human neurodevelopment and the specific cell types in the brain from ASD individuals that differentially express complement components are still largely unexplored. Human induced pluripotent stem cell (iPSC)-derived brain cells have been successfully used to model ASD [23,24,25,26,27] and we have recently shown that the transcriptome profiles from iPSC-derived neural progenitor cells (NPCs) and neurons best reflects neuronal tissue at early (4–10 post-conception weeks) and middle (16–24 post-conception weeks) stages of prenatal brain development, respectively [27]. This study aimed to characterize the constitutive mRNA and protein expression levels of a wide range of complement system components, receptors and regulators in neuronal (NPCs and neurons) and glial (astrocytes) cells derived from iPSCs of individuals with ASD and neurotypical controls, and to identify possible differences in complement expression between the ASD and control cells. 

## 2. Results

### 2.1. Generation of iPSC-Derived Neural Progenitor Cells, Neurons and Astrocytes from ASD and Control Subjects 

**Figure 1:**
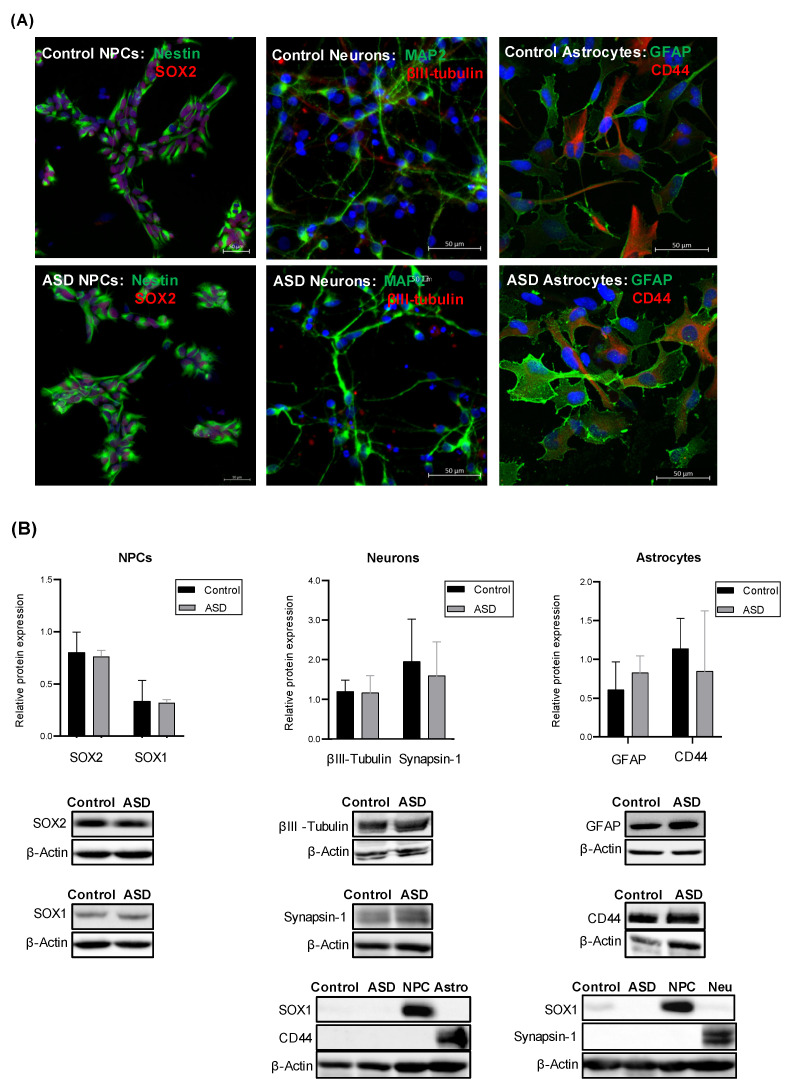
Expression of lineage-specific markers in iPSC-derived NPCs, neurons and astrocytes. (**A**) Representative images of control- and ASD-derived: NPCs, showing the expression of the neural progenitor markers Nestin, an intermediate filament protein, and SOX2, a transcription factor that regulates pluripotency; neurons showing the expression of the neuron-specific cytoskeleton proteins MAP2 and βIII-tubulin; astrocytes showing the expression GFAP, one of the major intermediate filament protein of astrocytes, and CD44, a cell adhesion protein expressed by astrocyte precursor cells. Nuclei were stained with DAPI (blue). All scale bars represent 50 μm. (**B**) Relative protein levels of: SOX2 and SOX1 in iPSC-derived NPCs of control (*n* = 4) and ASD (*n* = 7) subjects; βIII-tubulin and Synapsin-1, a synaptic vesicle-associated protein, in iPSC-derived neurons of control (*n* = 4) and ASD (*n* = 5) subjects; GFAP and CD44 in iPSC-derived astrocytes of control (*n* = 4) and ASD (*n* = 5) subjects. β-actin was used as a loading control. Representative immunoblot images of each lineage-specific marker are shown. No significant differences in the expression levels of these lineage markers were observed between the control and ASD groups. Furthermore, in order to verify whether the neuron cultures were contaminated by NPCs and astrocytes, as well as whether the astrocyte cultures were contaminated by NPCs and neurons, the expression of SOX1 and CD44 was measured in control- and ASD-derived neurons, and the expression of SOX1 and Synapsin-1 was measured in control- and ASD-derived astrocytes. NPC = iPSC-derived NPCs used as the control; Neuro = iPSC-derived neurons used as the control; Astro = iPSC-derived astrocytes used as the control. No detectable expression of SOX1 and CD44 was found in neurons, and no detectable expression of SOX1 and Synapsin-1 was found in astrocytes, suggesting that these cultures were not mixed neuronal/glial cultures.

iPSCs from individuals with ASD (*n* = 7) and control subjects (*n* = 4) were differentiated into NPCs, post-mitotic neurons and astrocytes, and the expression of lineage-specific markers was measured by western blotting and qualitatively confirmed by immunocytochemistry. We observed that NPCs expressed Nestin, SOX2 and SOX1, while neurons expressed MAP2, βIII-tubulin and Synapsin-1, and astrocytes expressed GFAP and CD44, which are typical markers of each of these cell types. Furthermore, no significant differences in the expression levels of these markers were observed between the ASD and the control group (Figure 1). As stem cell differentiation protocols might generate heterogeneous cell populations, and the proportion of different types of cells can vary from sample to sample, we also measured, by western blotting, the expression levels of SOX1 and CD44 in neurons and the expression of SOX1 and Synapsin-1 in astrocytes. We found no detectable expression of the markers for neural stem/progenitor cells and astrocytes in ASD and control neurons and no detectable expression of the markers for neural stem/progenitor cells and neurons in ASD and control astrocytes (Figure 1). These results suggest that these cultures, although not comprised of pure populations of specific neuronal and glial subtypes, appear not to be mixed neuronal/astrocyte cultures.

### 2.2. Expression of Complement Genes in iPSC-Derived Neural Progenitor Cells, Neurons and Astrocytes

**Figure 2:**
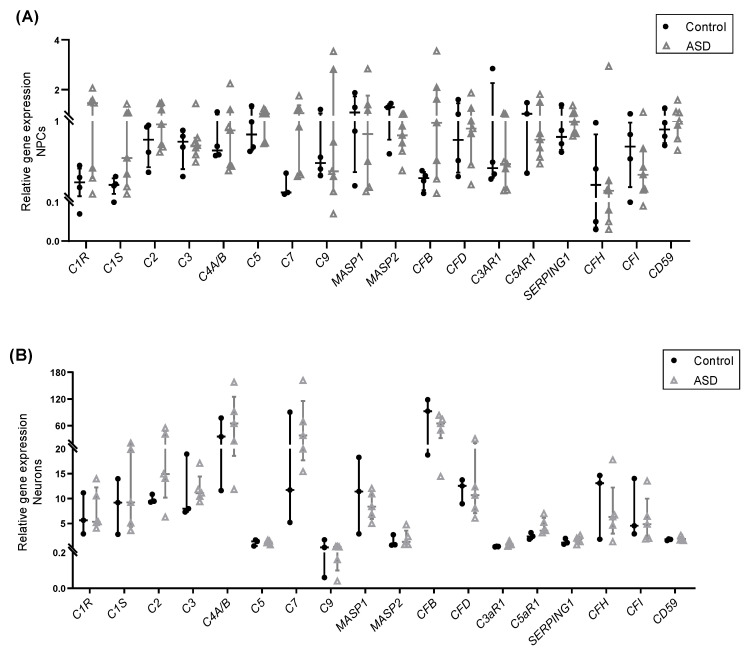

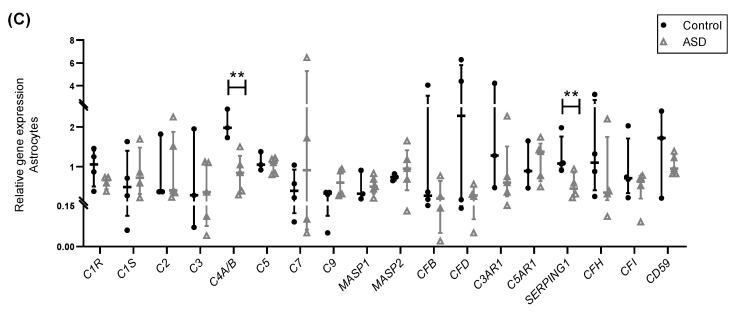
Expression of complement genes in iPSC-derived neural progenitor cells, neurons and astrocytes. Relative transcript levels of key complement system components, receptors, and regulators measured by RT-qPCR in: (**A**) iPSC-derived NPCs of control (*n* = 4) and ASD (*n* = 7) subjects; (**B**) iPSC-derived neurons of control (*n* = 3) and ASD (*n* = 5) subjects; (**C**) iPSC-derived astrocytes of control (*n* = 4) and ASD (*n* = 5) subjects. A significant decrease in the expression levels of *C4A/B* and *SERPING1* mRNAs was observed in astrocytes derived from individuals with ASD compared to control astrocytes. ** *p* < 0.01.

First, we examined the gene expression levels of several complement system components (*C1R*, *C1S*, *C2*, *C3*, *C4A/B*, C5, *C7*, *C9*, *MBL2*, *MASP1*, *MASP2*, *CFB*, *CFD*), receptors (*C3aR1* and *C5aR1*) and regulators (*SERPING1, CFH, CFI,* and *CD59*) in ASD- and control-derived NPCs, neurons and astrocytes by RT-qPCR. The results showed that, except for *MBL2*, mRNA transcripts for these key complement genes were detected in all the samples analyzed. Some genes exhibited highly variable expression across samples, and the expression values of most genes were significantly higher in neurons than in NPCs and astrocytes (Figure 2A–C; Figure S1A). No significant differences in mRNA expression levels were found between ASD and control NPCs, as well as between ASD and control neurons, which was in agreement with a previous transcriptomic study using the same iPSC-derived cells [27]. However, a larger sample size would be required to draw definite conclusions (power < 0.80). On the other hand, a significant decrease in the expression levels of *C4A/B* (paralogous *C4A* and *C4B* genes) (*p* < 0.01; power = 0.81) and *SERPING1* (*p* < 0.01; power = 0.80) was observed in astrocytes derived from ASD subjects compared to control astrocytes (Figure 2C). 

### 2.3. Expression of Complement Proteins in iPSC-Derived Neural Progenitor Cells, Neurons and Astrocytes

**Figure 3:**
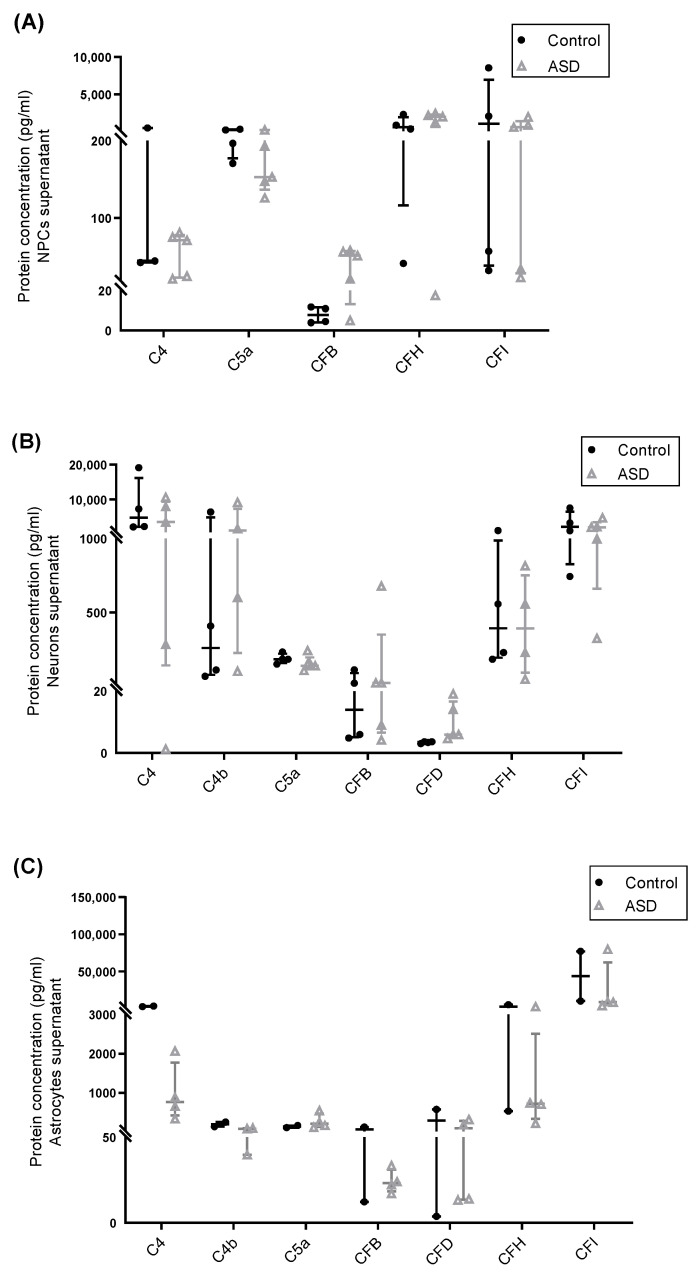

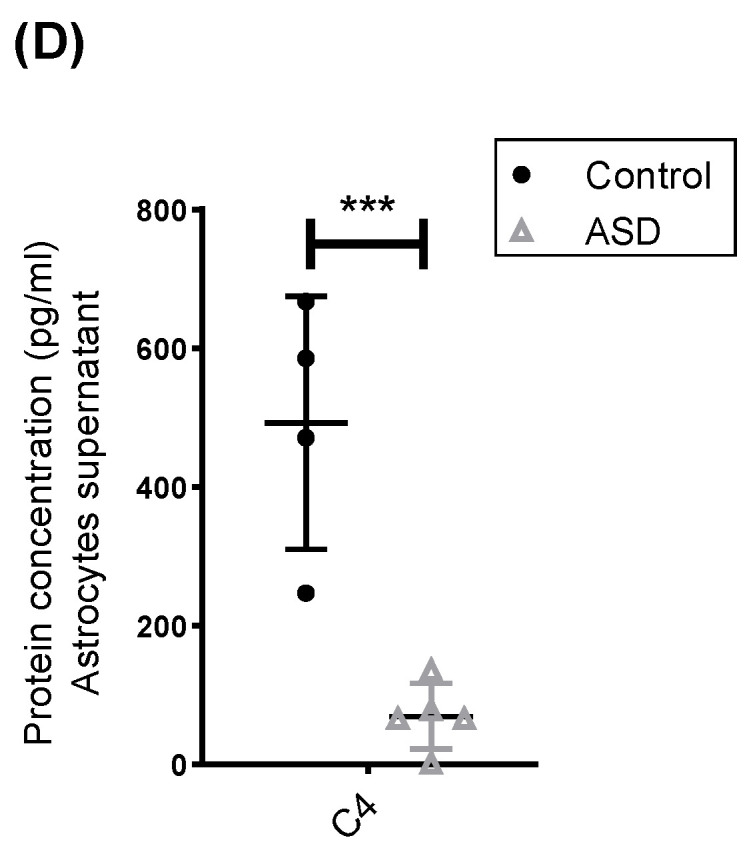
Secretion of complement proteins by iPSC-derived neural progenitor cells, neurons and astrocytes. Protein levels (pg/mL) of complement components and regulators measured by multiplex assays in culture supernatants from (**A**) iPSC-derived NPCs of control (*n* = 4) and ASD (*n* = 5) subjects; (**B**) iPSC-derived neurons of control (*n* = 4) and ASD (*n* = 5) subjects; (**C**) iPSC-derived astrocytes of control (*n* = 2) and ASD (*n* = 4) subjects. (**D**) Protein levels of C4 in culture supernatants of iPSC-derived astrocytes of control (*n* = 4) and ASD (*n* = 4) subjects were also assessed by using an ELISA assay. A significant decrease in the secretion levels of C4 was observed in astrocytes derived from individuals with ASD compared to control astrocytes. *** *p* < 0.001.

Next, protein levels of several soluble complement components (C1q, C2, C3, C3b/iC3b, C4, C4b, MBL, C5, C5a, CFB, CFD) and regulators (CFH and CFI) in culture supernatants from ASD- and control-derived NPCs, neurons and astrocytes were measured by multiplex assays. The results indicated that all cell samples secreted reliably detectable amounts (above the lower limit of detection of the multiplex assays) of C4, C5a, CFB, CFH and CFI, and that the expression levels of most of these proteins varied substantially across samples. Notably, neurons and astrocytes secreted significantly higher levels of C4 than NPCs, and detectable levels of C4b, a C4 cleavage product, were only observed in the supernatants of these cell types. Furthermore, astrocytes and neurons secreted reliably detectable levels of CFD (Figure 3A–C; Figure S1B). Protein levels of C1q, C2, C3, C3b/iC3b, C5, and MBL were below the lower limit of detection of the multiplex assays in culture supernatants from most samples and were not considered for further analysis. 

No significant differences in the secretion levels of complement proteins were found between ASD and control NPCs, neurons, and astrocytes, but the sample size was insufficient to firm conclusions (power < 0.80). However, we observed a tendency towards decreased secretion of C4 and C4b by ASD astrocytes compared to control astrocytes (Figure 3C), which was in accordance with the significant diminished *C4A/B* mRNA expression in these cells. In order to confirm the obtained results, the C4 protein level was also quantified in astrocyte culture supernatants by using a human C4 ELISA assay and a full set of independent experiments with a larger sample of astrocytes. The results revealed that ASD astrocytes under steady-state conditions indeed secreted significantly lower levels of C4 compared to control astrocytes (*p* < 0.001; power = 0.98) (Figure 3D). 

## 3. Discussion

In this study, we investigated the production of the main components of the three complement pathways and their key receptors and regulators in human iPSC-derived NPCs, neurons and astrocytes of ASD and control subjects, which are in vitro models that recapitulate some molecular and cellular aspects of the developing human brain. Although neurons and astrocytes comprise heterogeneous populations of cells in the brain and our cultures did not consist of pure populations of specific neuronal and astroglial cell types, no significant differences in the expression of typical cell markers were observed between the ASD and control groups, and we were able to find some consistent results which provided an important starting point for a better understanding of the role of the complements in neurodevelopment and how their dysfunction might contribute to ASD risk.

We observed that, while a large number of complement genes are transcribed in human NPCs, neurons and astrocytes, these cells under normal conditions may be an effective local source of only a specific set of complement components and regulators. We found that all ASD and control cell lines expressed mRNA transcripts for the *C5* and C5a receptor (*C5aR1*) and secreted C5a protein, produced from the cleavage of C5 (the levels of C5 protein fell below the assay lower limit of quantification and were not shown). Studies using mouse models have shown that C5a–C5aR1 signaling regulates NPC proliferation in the developing brain [4,28], and in vitro studies have shown that C5a and C5aR1 are constitutively expressed in human embryonic stem cells and iPSCs [29], NPCs [30], fetal astrocytes [31] and mouse cortical neurons [32,33], and they control key biological processes in these cells. Although we did not observe any differences in the expression patterns of C5a and C5aR1 between ASD and control neuronal and glial cells, our results corroborate the findings of previous studies showing expression and physiological roles of these complement molecules in different cell types in the developing human brain.

We also found that ASD and control NPCs, neurons and astrocytes secreted and/or expressed CFB and CFD, key components of the alternative complement pathway, and CFH and CFI, inhibitors of the classic and alternative complement pathways [1]. Although abnormal expression of these complement components and regulators has been implicated in cerebral inflammation [34], degeneration [35] and retinopathies [36,37], the possible roles of these proteins in ASD and in the normal developing human nervous system are still largely unknown.

Curiously, we observed that all the cell samples constitutively expressed *SERPING1* mRNA, and that the expression levels of this gene were significantly lower in astrocytes derived from ASD individuals compared to control astrocytes. *SERPING1* codes for a protease that binds to and inactivates C1r, C1s and MASP-1/2, thus leading to inhibition of the initial phases of classical and lectin pathways [38]. Because a *Serping1* deficiency leads to impaired NPC proliferation and neuronal migration in the developing mouse cerebral cortex [39], it would also be important to investigate the effects of reduced expression of human astrocyte-derived *SERPING1* on neurodevelopment and its relevance to the pathogenesis of ASD.

Finally, we observed constitutive expression and secretion of the critical component of both classical and lectin cascades C4 by ASD and control NPCs, neurons and astrocytes, as well as constitutive secretion of C4b by ASD and control neurons and astrocytes. Interestingly, we found that astrocytes derived from individuals with ASD expressed significantly lower levels of *C4A/B* mRNA and secreted significantly lower levels of C4 protein compared to control astrocytes. In the brain, C4 localizes to synapses and, together with other members of the classic complement cascade, has been shown to be required for synaptic elimination by microglia in the mouse developing visual system, as mice deficient for complement *C1q*, *C3*, *CR3* or *C4* exhibited impaired elimination of retinogeniculate synapses [6,7,8]. Accordingly, over-expression of *C4* in the mouse prefrontal cortex caused alterations in dendritic spine development, reduced connectivity, increased synaptic pruning and deficits in social behavior [9]. 

In humans, C4 is encoded by two different genes, *C4A* and *C4B*, which are located in tandem on the short arm of chromosome 6 and vary in copy number [8]. While *C4B* null alleles that decrease the expression of C4 have been associated with ASD [16,17,18], alleles at *C4A* that increase the expression of C4 have been associated with schizophrenia, and, mechanistically, it has been proposed that augmented C4 is involved in the exacerbated synaptic pruning and decreased synapse number in schizophrenic patients [8]. Therefore, taking all the above-mentioned data into account and the fact that other iPSC studies have reported abnormal neuronal connectivity of ASD neurons [23,24,25,26,27] and that astrocytes regulate synapse formation, elimination and activity [40,41,42], it is tempting to speculate that a reduced secretion of astrocyte-derived C4 might contribute to the reduced synaptic pruning and increased dendritic spine density in the brain of individuals with ASD [43,44]. However, additional studies are clearly needed to determine the consequences of decreased astrocyte-derived C4 for brain development and ASD pathophysiology, as well as to explore the C4 locus structure in ASD individuals.

In summary, our results provide insights into the expression patterns of a broad range of complement genes and proteins in iPSC-derived NPCs, neurons and astrocytes, and they revealed a decreased expression and secretion of complement C4 by astrocytes derived from ASD individuals. Furthermore, our findings highlight the use of human iPSC-derived neuronal and glial cells as effective platforms for the study of the complement system in human neurodevelopment.

## 4. Materials and Methods

### 4.1. Subjects and Genetic Analysis

All individuals evaluated in this study (*n* = 7 individuals with non-syndromic ASD and *n* = 4 neurotypical controls) were described previously [27,45,46,47]. CGH-array and whole exome sequencing using genomic DNA from the peripheral blood of the ASD subjects and their parents allowed the identification of known ASD pathogenic variants in two individuals: one patient harbored deleterious compound heterozygous variants in the *RELN* gene and a de novo splice site variant in the *CACNA1H* gene; the second patient harbored a duplication of 15q11–13. The remaining five patients did not harbor rare variants (minor allele frequency < 0.001) that cause a known deleterious loss of function (LoF) of an ASD gene (https://gene.sfari.org/; 16 June 2021). In addition, none the individuals with ASD harbored rare de novo LoF or missense variants with CADD-score ≥ 20 in the complement genes studied here (the full names and symbols of the complement genes are listed in Table S1).

### 4.2. Differentiation of Induced Pluripotent Stem Cells into Neural Progenitor Cells 

All iPSC samples used in this study have been previously generated and characterized [27,42]. For iPSC differentiation into NPCs, iPSCs were suspended to generate embryoid bodies and plated to produce neural rosettes, which were manually isolated, dissociated and cultured on dishes coated with 10 µg/mL poly-L-ornithine and 5 µg/mL laminin in NPC medium containing: DMEM/F12, 0.25X N2-supplement (Thermo Fischer Scientific, Waltham, MA, USA), 0.5X B27-supplement (Thermo Fischer Scientific), 20 ng/mL of FGF and 20 ng/mL EGF (Peprotech, Rocky Hill, NJ, USA). The cell culture medium was changed every other day. NPCs derived from either 1 or 2 iPSC clones of each subject were used in all experiments described herein.

### 4.3. Differentiation of Neural Progenitor Cells into Neurons

Neurons were produced from NPCs after 4 weeks of differentiation, as previously described [27]. Briefly, NPCs were seeded on plates coated with 20 µg/mL poly-l-ornithine and 10 µg/mL laminin. After reaching 50% confluence, cells were cultured with DMEM/F12 medium supplemented with 0.5X N2, 1X B27 and 1 μM of retinoic acid (Sigma-Aldrich, St. Louis, MO, USA), which was changed every three days. It is worth mentioning that by using this differentiation protocol and analyzing the expression profile of specific cell-type markers, we have previously shown that these iPSC-derived neuronal cultures were characterized by the presence of both GABAergic and glutamatergic neurons, with no evidence for the presence of dopaminergic and serotoninergic neurons [27].

### 4.4. Differentiation of Neural Progenitor Cells into Astrocytes

Astrocytes were differentiated from NPCs, as previously described [48]. Briefly, NPCs were mechanically detached and cultured in a suspension in ultra-low attachment plates with NPC medium, under agitation at 90 rpm to allow for 3D sphere formation. Twenty-four hours later, the medium was changed to DMEM/F12, supplemented with 0.5X N2, 0.5X B27 and 10 µM ROCK inhibitor, and spheres were maintained in suspension culture for 48 h. Subsequently, the medium was changed to Astrocyte Growth Medium (AGM Bullet Kit, Lonza, Walkersville, MD, USA), and spheres were maintained in suspension culture for an additional 15 days. The cell culture medium was changed every other day. The spheres were then seeded on plates coated with 10 µg/mL poly-l-ornithine and 5 µg/mL laminin and cultured in AGM until astrocytes spread from adherent spheres. When astrocytes reached confluency, spheres were manually removed, and astrocytes were passaged at least three times before usage in any experiment. 

### 4.5. Immunocytochemistry

Each cell line was seeded on cover slips coated with poly-l-ornithine and laminin. Cell monolayers were fixed with 4% paraformaldehyde (Sigma Aldrich), and after permeabilization with blocking buffer (5% donkey serum and 1% triton in PBS) at room temperature for 1 h, cells were incubated with the following primary antibodies diluted in blocking buffer overnight at 4 °C: anti-SOX2 (1:100, AB5603; Millipore, Billerica, MA, USA); anti-Nestin (1:250, MAB5326; Millipore); anti-MAP2 (1:500, M9942; Sigma-Aldrich); anti-βIII-tubulin (1:2000, MMS435P; Covance, Princeton, NJ, USA); anti-GFAP (1:1000, AB5804; Millipore); anti-CD44 (5 μg/mL, ab6124; Abcam, Cambridge, MA, USA). Subsequently, cells were washed with PBS and incubated at room temperature for 1 h, with secondary antibodies conjugated with AlexaFluor594 or AlexaFluor488 (1:400; Thermo Fisher Scientific). Nuclei were stained with DAPI (VECTASHIELD, Vector Laboratories). Fluorescence images were obtained using a Zeiss LSM 710 confocal microscope system (Carl Zeiss Meditec AG, Jena, Germany) and used as an initial qualitative test. 

### 4.6. Protein Extraction and Immunoblotting

Total protein extracts from each cell line were obtained using a Ripa Buffer containing protease and phosphatase inhibitor cocktails (Sigma Aldrich) and quantified using a BCA assay (Thermo Fisher Scientific). For immunoblotting, total proteins (20 μg) were separated by SDS–PAGE and transferred to nitrocellulose membranes (GE HealthCare, Chicago, IL, USA), which were then blocked and incubated overnight at 4 °C with the following primary antibodies: anti- SOX2 (1:1000, AB5603; Millipore); anti-SOX1 (1:1000, #4194; Cell Signaling Technology, Danvers, MA, USA); anti-βIII-tubulin (1:2000, MMS435P; Covance); anti-Synapsin-1 (1:1000, #5297; Cell Signaling Technology); anti-CD44 (1:1000, ab6124; Abcam); anti-GFAP (1:1000, AB5804; Millipore); anti-β-actin (1:10,000, A2228; Sigma Aldrich), for loading control. In addition, the following antibodies were used against C3aR1, C5aR1 and SERPING1 proteins and produced multiple nonspecific bands on western blots using total protein from NPC, neuron or astrocyte lysates: ab126250, ab59390 and ab134918 (Abcam), respectively. Detection was performed using horseradish peroxidase-coupled anti-rabbit or anti-mouse secondary antibodies (1:2000, #7074 or #7072; Cell Signaling Technology), ECL substrate (Bio-Rad, Hercules, CA, USA) and the ChemiDoc MP Imaging System (Bio-Rad). The intensity of the bands was determined by densitometry, using The Image Lab Software (Bio-Rad). The graphs show the relative expression levels of each protein (normalized to β-actin) for each group (Control and ASD) with mean and SD, and they combine results from two independent experiments with similar results.

### 4.7. RNA Extraction and Quantitative Real-Time PCR

Total RNA from each cell line was extracted using the NucleoSpin^®^ RNA kit (Macherey-Nagel, Dueren, Germany), following the manufacturer’s instructions. RNA quality and quantity were assessed using the NanoDrop 3300 Fluorospectrometer (Thermo Fisher Scientific). The cDNA was synthesized from 500 ng of total RNA with the SuperScript III First-Strand Synthesis System (Thermo Fisher Scientific). The qPCR reactions were performed in triplicate with 60 ng of cDNA and predesigned TaqMan gene expression assays (Table S1) in a QuantStudio 6 Flex Real-Time PCR System (Applied Biosystems, Foster City, CA, USA). The expression levels of 20 target genes of the complement system (*C1R*, *C1S*, *C2*, *C3*, *C4A/B*, *C5*, *C7*, *C9*, *MBL2*, *MASP1*, *MASP2*, *CFB*, *CFD*, *C3aR1*, *C5aR1*, *SERPING1*, *CFH*, *CFI* and *CD59*) were normalized to the mean expression value of the *GAPDH* and *HMBS* housekeeping genes. It is noteworthy that the qPCR assay used detects the highly homologous *C4A* and *C4B* genes. Results are expressed as the mean fold change of the normalized gene expression relative to a calibrator sample using the comparative CT method (2^−ΔΔ*C*t^ method). The dot-plots show the transcript levels for each individual in the Control and ASD groups and combine results from two independent experiments with similar results. All ASD and control samples were processed simultaneously, and measurements were taken during the same experimental run, which was performed using only high-quality mRNA samples.

### 4.8. Multiplex and ELISA Assays

Cells were cultured until 90% confluence in 60 mm tissue culture plates, and the supernatants were collected after 48h cultures and centrifuged to remove cell debris. Cell-free supernatants (2.5 mL) were then transferred to a new conical tube, and the proteins in the supernatants were precipitated by adding ice-cold acetone (4:1) and resuspended in 250 μL of RIPA buffer containing protease inhibitors (10X concentrated supernatants). The concentration of 13 complement components and regulators were quantified in cell culture supernatants by using the Luminex xMAP detection system and the Human Complement Panel 1 and 2 Bead-Based Multiplex Assay kits (HCMP1MAG-19K and HCMP2MAG-19K, Millipore), whose detection sensitivities are (minimum detectable concentrations) C2 = 310 pg/mL, C4b = 360 pg/mL, C5 = 1040 pg/mL, C5a = 5.1 pg/mL, CFD = 26pg/mL, MBL = 40 pg/mL, CFI = 220 pg/mL, and C1q = 48 pg/mL, C3 = 120 pg/mL, C3b/iC3b = 3639 pg/mL, C4 = 191 pg/mL, CFB = 24 pg/mL and CFH = 135 pg/mL respectively. All samples were assayed in duplicate. Data acquisition and analyses were performed using the Luminex^®^ xPONENT^®^ and the Milliplex^®^ Analyst 5.1 softwares (Millipore), respectively. Values below the minimum detectable concentrations of the multiplex assays were not considered. C4 protein levels were also quantified in astrocyte culture supernatants in a full set of independent experiments (including novel astrocyte differentiation, protein extraction and quantification) by using an ELISA assay (Human Complement C4 ELISA kit, ab108825, Abcam), following the manufacturer’s instructions. Optical density measurements were taken at 450 nm using a GloMax^®^ Discove Microplate Reader (Promega, Madison, WI, USA). All test samples were analyzed in duplicate and fell above the minimum detectable concentration of the ELISA assay (41 pg/mL). Results were normalized to the total protein content of the cell lysates and were expressed in pg/mL. The dot-plots show the protein levels for each individual in the Control and ASD groups and combine results from two independent experiments with similar results. All ASD and control samples were processed simultaneously, and measurements were taken during the same experimental run, which was performed using only high-quality samples.

### 4.9. Statistical Analysis

Statistical analyses were carried out using the generalized linear mixed-effect model in SPSS software to account for dependency between biological replicates (when NPCs, neurons and astrocytes derived from different iPSC clones of the same individual were used) and independent technical replicates per individual. *p* values < 0.05 were considered statistically significant. Data obtained by RT-qPCR, multiplex and ELISA analysis were represented as the median with the interquartile range. The statistical power calculation was conducted using the PASS (Power Analysis and Sample Size) software, and power values of 0.80 or greater were considered appropriate to reject the null hypothesis of zero correlation.

## Data Availability

All raw data are available from the corresponding author on reasonable request.

## Supplementary Materials

The following are available online at https://www.mdpi.com/article/10.3390/ijms22147579/s1.
