# Supplementary material for: Complement C4 Is Reduced in iPSC-Derived Astrocytes of Autism Spectrum Disorder Subjects
Source: Int J Mol Sci. 2021 Jul 15;22(14):7579. doi: 10.3390/ijms22147579 (PMC8305914; doi:10.3390/ijms22147579)
Supplement: Supplementary file 1 [file ijms-22-07579-s001.zip › ijms-1265110-supplementary.pdf]

Supplementary Figure S1

(A)

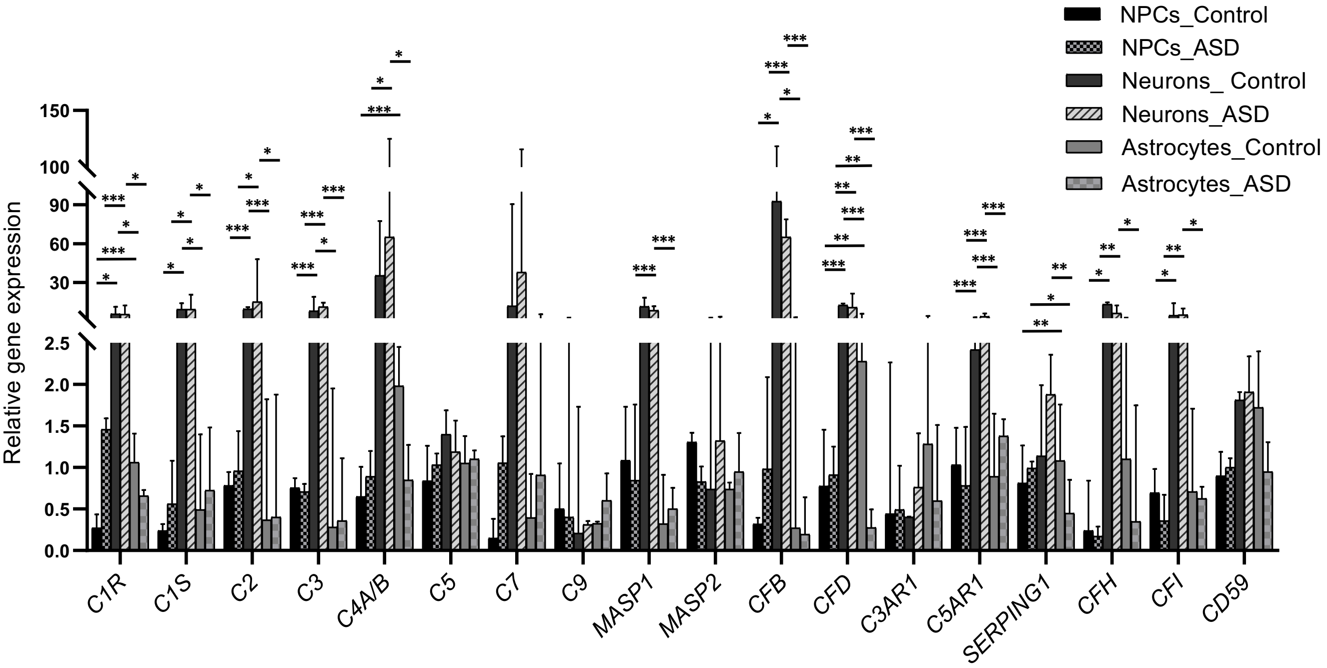

(B)

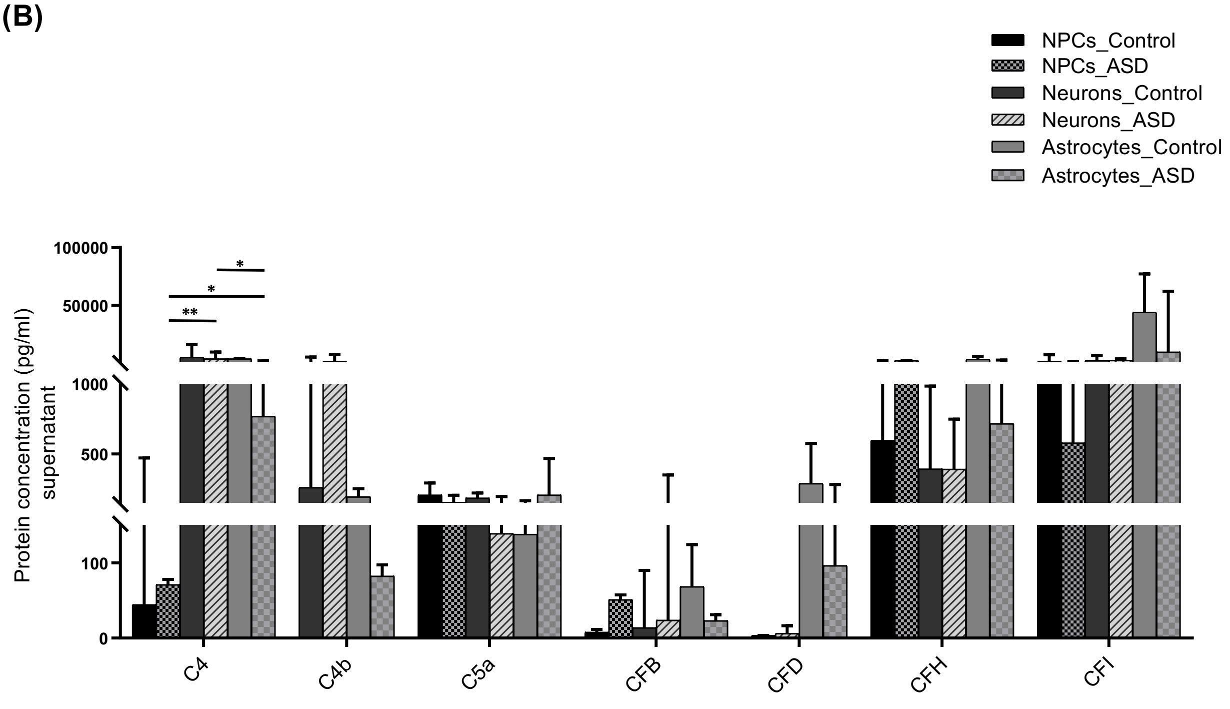

Figure S1. Comparison of the expression levels of complement genes and proteins between iPSC-derived neural progenitor cells and iPSC-derived neurons, iPSC-derived neural progenitor cells and iPSC-derived astrocytes, and iPSC-derived neurons and iPSC-derived astrocytes. (A) Relative transcript levels of key complement system components, receptors, and regulators measured by RT-qPCR in iPSC-derived NPCs of control (n=4) and ASD (n=7) subjects, iPSC-derived neurons of control (n=3) and ASD (n=5) subjects, iPSC-derived astrocytes of control (n=4) and ASD (n=5) subjects. **(B)** Protein levels (pg/ml) of complement components and regulators measured by multiplex assays in culture supernatants from iPSC-derived NPCs of control (n=4) and ASD (n=5) subjects, iPSC-derived neurons of control (n=4) and ASD (n=5) subjects, iPSC-derived astrocytes of control (n=2) and ASD (n=4) subjects. *p< 0.05; **p< 0.01; ***p< 0.001.

Supplementary Table S1

Supplementary Table 1: List of the complement genes studied and the predesigned TaqMan gene expression assays used for qPCR.

Gene symbol	Full name	TaqMan Assay ID
**Complement genes**		
C1R	Complement C1r	Hs00354278_m1
C1S	Complement C1s	Hs00156159_m1
C2	Complement C2	Hs00918862_m1
C3	Complement C3	Hs00163811_m1
C4A, C4B	Complement C4A, C4B	Hs00246758_m1
C5	Complement C5	Hs01004342_m1
C7	Complement C7	Hs00940408_m1
C9	Complement C9	Hs01036216_g1
MBL2	Mannose Binding Lectin 2	Hs00175093_m1
MASP1	MBL Associated Serine Protease 1	Hs00373559_m1
MASP2	MBL Associated Serine Protease 2	Hs01548237_g1
CFB	Complement Factor B	Hs00156060_m1
CFD	Complement Factor D	Hs00157263_m1
C3AR1	Complement 5a Receptor 1	Hs00269693_s1
C5AR 1	Complement 5a Receptor 2	Hs00704891_s1
SERPING1	Serpin Family G Member 1	Hs00163781_m1
CFH	Complement factor I	Hs00962373_m1
CFI	Complement factor H	Hs00989715_m1
CD59	Cluster of differentiation 59	Hs00174141_m1
**Housekeeping gene**		
GAPDH	Glyceraldehyde-3-Phosphate Dehydrogenase	Hs02758991_g1;
HMBS	Hydroxymethylbilane Synthase	Hs00609296_g1
